# CEACAM5, KLK6, SLC35D3, POSTN, and MUC2 mRNA Analysis Improves Detection and Allows Characterization of Tumor Cells in Lymph Nodes of Patients Who Have Colon Cancer

**DOI:** 10.1097/DCR.0000000000002151

**Published:** 2021-06-23

**Authors:** Lina M. Olsson, Gudrun E. Lindmark, Anne C.E. Israelsson, Dejan Korkocic, Sten G. Hammarström, Marie-Louise K.C. Hammarström

**Affiliations:** 1 Department of Clinical Microbiology, Section of Infection and Immunology, Umeå University, Umeå, Sweden; 2 HiloProbe AB, Umeå, Sweden; 3 Department of Clinical Sciences, Lund University, Helsingborg, Sweden; 4 Specialistläkarna, Malmö, Sweden; 5 Department of Pathology, Helsingborg Hospital, Helsingborg, Sweden

**Keywords:** Biomarker mRNA expression levels, Carcinoembryonic antigen, Hematoxylin-eosin staining, Lymph node metastasis, Side-by-side comparison

## Abstract

Lymph node metastasis is the single most important prognostic risk factor for recurrence in patients with colon cancer who have undergone curative surgery. The routine method for detecting disseminated tumor cells in lymph nodes is microscopic examination of one or a few hematoxylin and eosin-stained tissue sections by a trained pathologist. This method, however, is insensitive mainly because less than 1% of the lymph node volume is examined, leading to misclassification.

**OBJECTIVE:**

This study aimed to investigate whether analysis of a selected group of biomarker mRNAs improves detection and characterization of lymph node metastases/micrometastases compared with the routine method.

**DESIGN:**

This study is a side-by-side comparison of biomarker mRNA analysis and histopathology of 185 lymph nodes from patients with colon cancer representing stages I to IV, and an investigation of the importance of lymph node tissue volume for tumor cell detection.

**SETTINGS:**

This is a collaborative study between a high-volume central hospital and a preclinical university institution.

**PATIENTS:**

Fifty-seven patients who had undergone tumor resection for colon cancer were included.

**MAIN OUTCOME MEASURES:**

The primary outcomes measured were mRNA copies per 18S rRNA copy of CEACAM5, KLK6, SLC35D3, POSTN, and MUC2 by multiplex assay and metastases/micrometastases detected by histopathology.

**RESULTS:**

The number of tumor cell-positive lymph nodes was 1.33-fold higher based on CEACAM5 mRNA levels compared with histopathological examination. Increasing the tissue volume analyzed for CEACAM5 levels from an 80-µm section to half a lymph node increased the number of positive nodes from 34 of 107 to 80 of 107 (*p* < 0.0001). Similarly, the number of positive nodes for the aggressiveness marker KLK6 increased from 9 of 107 to 24 of 107.

**LIMITATIONS:**

Only a limited number of individual lymph nodes per patient was available for analysis.

**CONCLUSIONS:**

mRNA analysis of CEACAM5, KLK6, and SLC35D3 improves the detection of tumor cells in lymph nodes from patients surgically treated for colon cancer, and, together with POSTN and MUC2, it further allows characterization of the tumor cells with respect to aggressiveness and the tumor cell environment. See **Video Abstract** at http://links.lww.com/DCR/B650.

**EL ANÁLISIS DE ARNM DE CEACAM5, KLK6, SLC35D3, POSTN Y MUC2 MEJORA LA DETECCIÓN Y PERMITE LA CARACTERIZACIÓN DE CÉLULAS TUMORALES EN LOS GANGLIOS LINFÁTICOS DE PACIENTES CON CÁNCER DE COLON:**

**ANTECEDENTES:**

Las metástasis en los ganglios linfáticos son el factor de riesgo pronóstico más importante de recurrencia en pacientes con cáncer de colon que se han sometido a cirugía curativa. El método de rutina para detectar células tumorales diseminadas en los ganglios linfáticos es el examen microscópico de una o algunas secciones de tejido teñidas con hematoxilina-eosina por un patólogo capacitado. Sin embargo, este método es insensible principalmente porque se examina menos del 1% del volumen de los ganglios linfáticos, lo que conduce a una clasificación errónea.

**OBJETIVO:**

Investigar si el análisis de un grupo seleccionado de ARNm de biomarcadores mejora la detección y caracterización de metástasis / micrometástasis en los ganglios linfáticos en comparación con el método de rutina.

**DISEÑO:**

Una comparación en paralelo del análisis de ARNm de biomarcadores y la histopatología de 185 ganglios linfáticos de pacientes con cáncer de colon que representan las etapas I-IV, e investigación de la importancia del volumen de tejido de los ganglios linfáticos para la detección de células tumorales.

**ENTORNO CLINICO:**

Estudio colaborativo entre un hospital central de alto volumen y una institución universitaria preclínica.

**PACIENTES:**

Cincuenta y siete pacientes que han sido sometidos a resección tumoral por cáncer de colon.

**PRINCIPALES MEDIDAS DE VALORACION:**

copias de ARNm / copia de ARNr 18S de CEACAM5, KLK6, SLC35D3, POSTN y MUC2 mediante análisis múltiple y metástasis / micrometástasis detectadas por histopatología.

**RESULTADOS:**

El número de ganglios linfáticos con células tumorales positivas fue 1,33 veces mayor según los niveles de ARNm de CEACAM5 en comparación con el examen histopatológico. El aumento del volumen de tejido analizado para los niveles de CEACAM5 de una sección de 80 µm a la mitad de un ganglio linfático aumentó el número de ganglios positivos de 34/107 a 80/107 (*p* <0,0001). De manera similar, el número de nodos positivos para el marcador de agresividad KLK6 aumentó de 9/107 a 24/107.

**LIMITACIONES:**

Solo un número limitado de ganglios linfáticos individuales / paciente estuvo disponible para el análisis.

**CONCLUSIONES:**

El análisis de ARNm de CEACAM5, KLK6 y SLC35D3 mejora la detección de células tumorales en los ganglios linfáticos de pacientes con cáncer de colon tratados quirúrgicamente y, junto con POSTN y MUC2, permite además la caracterización de las células tumorales con respecto a la agresividad y el entorno celular tumoral. Consulte **Video Resumen** en http://links.lww.com/DCR/B650.

## Abstract 2

Supplemental Digital Content is available in the text.

The curative treatment for colorectal cancer (CRC) is surgery. However, recurrence is common and regional lymph node (LN) metastasis is the single most important prognostic risk factor.^1–3^ Twenty-five percent of patients with metastasis-negative LNs (TNM stage I and II) will experience recurrence.^4,5^ Lymph node metastasis is routinely detected by microscopic examination of one or a few hematoxylin-eosin (H&E)-stained LN sections. This method has poor sensitivity, most likely because less than 1% of the LN volume is examined.^6,7^ In addition, the obvious risk of missing LN metastases (>2 mm in diameter), of importance for the decision on adjuvant chemotherapy, is accompanied by the even greater risk of missing micrometastases (clusters of 20 or more cells or 0.2–2.0 mm in diameter), pN1(mi), and isolated tumor cells (clusters with up to 20 tumor cells or <0.2 mm in diameter), pN0(i+).^8,9^ One way to increase the analyzed volume without excessively increasing the workload is to quantify biomarker mRNAs as proxies for tumor cells. We previously showed that CEACAM5 mRNA is expressed at high levels in tumor cells and not detected in immune cells, and that CEACAM5 mRNA levels are proportional to the number of disseminated tumor cells.^10,11^ Furthermore, high levels of CEACAM5 mRNA in LNs is an indicator of poor prognosis,^10–12^ and, when analyzed in combination with the 4 biomarker mRNAs KLK6, SLC35D3, POSTN, and MUC2, can identify patients at risk of recurrence with higher sensitivity than H&E.^12–14^ Except for MUC2, the latter biomarkers were identified by genome-wide hybridization bead array analysis by individually comparing the gene expression of 4 H&E(+) LNs and 3 primary tumors from 3 patients with stage III colon cancer (CC) against the gene expression of a panel of control tissues including LNs of 4 control patients and normal colon epithelial cells.^14^ Mucinous adenocarcinoma in CRC has better prognosis than adenocarcinoma, in general, and a high MUC2:CEACAM5 ratio in LNs is a sign of good prognosis.^12,15^ Combined in a formula, these biomarkers allow the allocation of patients with CRC to different risk categories with respect to recurrence, and biomarker risk category was shown to be an independent prognostic factor to TNM stage and tumor grade.^14^ Thus, in both metastasis-negative and metastasis-positive scenarios, this tumor biomarker combination may provide improved information about the risk of recurrence.

Here, we investigate whether analysis of CEACAM5 mRNA in combination with KLK6, SLC35D3, POSTN, and MUC2 mRNAs improves detection and characterization of LN metastases/micrometastases compared with the routine method by performing a side-by-side comparison between histopathology and the quantification of mRNA levels in adjacent LN sections. Furthermore, we addressed the question whether analyzing a larger part of the LN volume improves detection of aggressive tumor cells by comparing detection of CEACAM5, KLK6, and SLC35D3 mRNAs in the entire volume of half a LN with the small volume of 8 consecutive 10-µm sections from the other half of the LN.

## MATERIALS AND METHODS

### Lymph Nodes and Study Design

**FIGURE 1.**
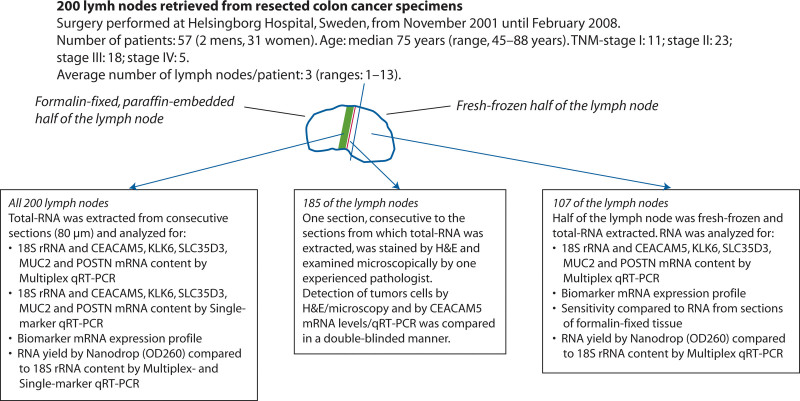
Study design and description of the clinical material. H&E = hematoxylin-eosin; OD260 = optical density at 260 nm; qRT-PCR = quantitative reverse transcription polymerase chain reaction.

Figure 1 summarizes the study design and characteristics of the patients with CC. Two hundred LNs were retrieved from the resected specimens of patients in whom a locally radical tumor resection for CC was performed. Lymph nodes were bisected with separate knives by the surgeon in the operating room. One-half of each LN was fixed in 10% formalin, embedded in an individual paraffin block, and archived at the Department of Pathology, Helsingborg Hospital, Sweden. These LN halves constitute a subgroup of the LNs used in a routine histopathology examination for pN classification for which a median of 14 (interquartile range (IQR): 10–19) LNs per patient was examined. Nine consecutive sections, 1 for H&E staining and 8 for RNA extraction (=80-µm section), were collected from these formalin-fixed halves of 200 LNs (33, 77, 65, and 25 LNs from 11 stage I, 23 stage II, 18 stage III, and 5 stage IV patients) with a median of 3 (IQR: 1–5) LNs per patient. Direct comparison between histopathology and CEACAM5 mRNA levels was performed in 185 LNs from 56 patients (31, 72, 60, and 22 LNs from 11 stage I, 23 stage II, 17 stage III, and 5 stage IV patients), with a median of 2 (IQR: 1–5) LNs per patient. Fifteen LNs were excluded for technical reasons (see description of histopathological examination below).

The other half of the LNs were snap-frozen in liquid nitrogen and stored at –70°C until RNA extraction. For the comparison between half-LN and 80-µm section RNA extracts, 107 paired extracts were analyzed. These LNs were from 30 patients (18, 40, 36, and 13 LNs from 3 stage I, 13 stage II, 11 stage III, and 3 stage IV patients), with a median of 3 (IQR: 1–6) LNs per patient.

Lymph nodes were given individual codes that were different for RNA extracted from sections, RNA extracted from fresh-frozen LN halves, and H&E-stained sections. Analyses were performed in a double-blinded manner.

### RNA Preparation

Total RNA was extracted from the formalin-fixed, paraffin-embedded 80-µm sections by using the RNeasy FFPE Kit (Qiagen, Sollentuna, Sweden) with minor modifications (Supplement http://links.lww.com/DCR/B649). Total RNA was extracted from the fresh-frozen half-LNs by using the acid guanidine phenol chloroform method, as described.^14^ RNA was stored at –70°C in RNase-free water containing RNasin ribonuclease inhibitor (Promega, Madison, WI).

### Quantification of RNA and 18S rRNA

Concentrations of RNA in the extracts were determined by measuring optical density at 260 nm (OD260), OD280, and OD230 using a NanoDrop ND-2000 spectrophotometer V1.4.1 (Thermo-Fisher-Scientific, Waltham, MA). Undiluted samples were used for RNA extracted from 80-µm sections and 1:10 dilutions for half-LN RNA extracts. The average yield of total RNA was 2.9 µg from the 80-µm section RNA extracts (n = 200) and 3574 µg from the half-LN extracts (n = 107; Table S1 http://links.lww.com/DCR/B649). Purity, estimated as OD260/OD280 ratio, was 1.95 (IQR: 1.92–1.99) for the 80-µm section extracts and 2.06 (IQR: 2.03–2.10) for the half-LN extracts.

Concentrations of 18S rRNA in RNA extracts were determined using a ColoNode Multiplex qRT-PCR kit (HiloProbe, Umeå, Sweden) and an in-house constructed single-marker, real-time qRT-PCR assay with RNA copy standard described in Table S2 http://links.lww.com/DCR/B649. ColoNode is composed of 2 triplex assays of which 1 measures 18S rRNA. The 80-µm section RNA extracts were analyzed undiluted by multiplex qRT-PCR and diluted 1:100 by single-marker qRT-PCR. The half-LN RNA extracts were analyzed at 1:10 dilution by the multiplex qRT-PCR assay only. There was a strong and highly significant correlation between the concentrations of total RNA determined by OD260 and 18S rRNA determined by qRT-PCR. In the 80-µm section RNA extracts, the correlation coefficient (*r*) values were 0.860 and 0.802 between total RNA and 18S rRNA determined by single-marker and multiplex qRT-PCR (*p* < 0.0001, n = 200; Figs. S1 A and C http://links.lww.com/DCR/B649). Similarly, there was strong correlation between the concentrations of total RNA and 18S rRNA in the half-LN RNA extracts (*r* = 0.834, *p* < 0.0001, n = 107; Fig. S1 D http://links.lww.com/DCR/B649).

The average yield of 18S rRNA in 80-µm section RNA extracts was 1.43 × 10^10^ and 3.13 × 10^10^ copies as determined by single-marker and multiplex qRT-PCR (Table S1 http://links.lww.com/DCR/B649). The yield of 18S rRNA in half-LN extracts was approximately 3 orders of magnitude higher (average 1.09 × 10^13^ copies), which is compatible with the average 1230 -fold higher yield of total RNA. 18S rRNA qRT-PCR also gave significant signals in samples with very low levels of total RNA measured as OD260 (Figs. S1 A, C, and D http://links.lww.com/DCR/B649). There was excellent correlation between 18S rRNA concentrations obtained by the multiplex and single-marker qRT-PCR (*r* = 0.918; Fig. S1 B http://links.lww.com/DCR/B649) and the 2 assays gave very similar estimates of total yield of 18S rRNA in the samples (Table S1 http://links.lww.com/DCR/B649).

### Gene Expression Analysis by Real-Time qRT-PCR

Quantification of mRNAs for CEACAM5, KLK6, SLC35D3, POSTN, and MUC2 was done in total RNA using the ColoNode Multiplex qRT-PCR kit and in-house constructed single-marker qRT-PCR assays with RNA copy standards. ColoNode is composed of 2 triplex assays that are run simultaneously, 1 reaction measuring CEACAM5, KLK6, and SLC35D3 and 1 reaction measuring POSTN, MUC2, and 18S rRNA. Analyses by multiplex qRT-PCR were performed in duplicate, and analyses by single-marker qRT-PCR were performed in triplicate. RNA extracts of the 80-µm sections were analyzed undiluted and half-LN extracts at 1:10 dilution. Table S2 http://links.lww.com/DCR/B649 describes the single-marker qRT-PCR assays for CEACAM5, KLK6, and MUC2 mRNAs. The assays for SLC35D3 and POSTN mRNAs were described previously.^14^ Results are expressed as concentration of mRNA copies/µL after correction for dilution or expressed as mRNA level normalized to the amount of 18S rRNA in the sample (mRNA copies per 18S rRNA copy). All qRT-PCR analyses were performed with samples containing >2.9 × 10^6^ 18S rRNA copies, with medians of 4.2 × 10^9^ (IQR: 1.8 × 10^9^ to 7.9 × 10^9^) and 5.6 × 10^10^ (IQR: 3.2 × 10^10^ to 8.3 × 10^10^) 18S rRNA copies per reaction for 80-µm section and half-LN RNA extracts.

A highly significant correlation (*p* < 0.0001) was seen between concentrations determined by multiplex and single-marker qRT-PCR for all 5 biomarker mRNAs. In 80-µm section extracts, the *r* values were 0.825, 0.942, 0.759, 0.934, and 0.734 for CEACAM5, KLK6, SLC35D3, POSTN, and MUC2 (n = 200; Fig. S2 http://links.lww.com/DCR/B649). POSTN was detected in all LNs by the single-marker assay and in all except 3 by the multiplex assay (Fig. S2 http://links.lww.com/DCR/B649). There was good congruence between results obtained by multiplex and single-marker assays also in half-LN extracts (Fig. S3 http://links.lww.com/DCR/B649). The multiplex assay was slightly less sensitive than the corresponding single-marker assays for CEACAM5, SLC35D3, and MUC2 mRNAs with detection limits in the multiplex assay of 100, 1000, and 10 mRNA copies/µL. All 5 biomarker mRNAs showed a strong correlation between the multiplex and single-marker assays in samples with concentrations above the detection limit of the multiplex assay (*r* > 0.9; Table S3 http://links.lww.com/DCR/B649). No sample was positive in the multiplex assay only.

### Histopathological Examination

The first of 9 consecutive sections from 200 formalin-fixed paraffin-embedded LNs was stained with H&E, prepared for microscopy according to the clinical routine procedure, and all were evaluated by an experienced pathologist (D.K.) for the presence of metastases >2.0 mm in diameter and micrometastases ≤2.0 mm in diameter grouped together with aggregated and isolated tumor cells. Fourteen LNs were excluded because the microscopy slides were empty, which excluded side-by-side comparison with RNA extracts, and 1 LN was excluded because the slide had primary tumor deposit.

### Statistical Analysis

Correlation analyses were performed using 2-sided Spearman rank correlation test. Differences in biomarker mRNA concentrations and levels between 2 groups were analyzed by 2-tailed Mann-Whitney *U* test. Significance of differences in numbers of LNs with detected CEACAM5 mRNA by analysis of half-LN compared to 80-µm section RNA extracts was analyzed by using the Fisher exact test. A *p* value <0.05 was considered statistically significant. Descriptive values are given as median and IQR. GraphPad Prism 6 (GraphPad Software, San Diego, CA) was used for statistical calculations.

### Ethics Statement

All procedures involving human participants were performed in accordance with the ethical standards of the institutional research committee and with the Helsinki Declaration. Lymph nodes were collected after patients’ written, informed consent. The study was approved by the Local Ethics Research Committee of the Medical Faculty, Umeå University, Umeå, Sweden (Registration number: 03-503). Access to archived samples was granted by Regional Biobank Center, Southern Healthcare Region, Lund, Sweden (Dnr 2015-A-68).

## RESULTS

### Biomarker Expression Profiles in Individual LNs

Expression levels of CEACAM5, KLK6, SLC35D3, POSTN, and MUC2 mRNAs were determined in 80-µm section RNA extracts of 200 LNs from 57 patients with CC by multiplex qRT-PCR. CEACAM5 mRNA was detected in 28.5% of the LNs, as a single biomarker in 13% of the LNs and in combination with other biomarkers in the residual 15.5%. POSTN was the only biomarker detected in CEACAM5-negative LNs, and 7.5% of the LNs were single positive for POSTN with levels above the cutoff for prognostic value (Table 1). Of note, only 7 of the 22 possible biomarker combinations were expressed in LNs (Table 1). The most frequent combination was CEACAM5/KLK6/SLC35D3/POSTN (5.5%) followed by CEACAM5/POSTN and CEACAM5/KLK6/POSTN.

**TABLE 1.** Biomarker expression profiles in 200 individual LNs of patients with colon cancer

Biomarker profile expressed by the LN^a^	No. of LNs^b^	%^c^
None of the 5 biomarkers	128	64.0
CEACAM5	26	13.0
POSTN	15	7.5
CEACAM5/KLK6/SLC35D3/POSTN	11	5.5
CEACAM5/POSTN	7	3.5
CEACAM5/KLK6/POSTN	6	3.0
CEACAM5/MUC2	3	1.5
CEACAM5/KLK6/POSTN/MUC2	2	1.0
CEACAM5/KLK6	1	0.5
CEACAM5/KLK6/MUC2	1	0.5

LN = lymph node; qRT-PCR, quantitative reverse transcription polymerase chain reaction.

a CEACAM5, KLK6, SLC35D3, POSTN, and MUC2 mRNA levels were determined in 80-µm section RNA extracts of 200 LNs by multiplex qRT-PCR. Lymph nodes were considered positive for POSTN when the level was above the 80th percentile, ie, 5.52 × 10^–6^ mRNA copies/18S rRNA copy, which is the cutoff for POSTN as an indicator for bad prognosis.^14^ Lymph nodes were considered positive for MUC2 when the value of MUC2 mRNA copies/CEACAM5 mRNA copy was >0.06, which is the cutoff for MUC2 as an indicator for good prognosis.^12^

b Number of LNs positive for the indicated biomarker mRNA(s).

c Percentage of the 200 investigated LNs that were positive for the indicated biomarker mRNA(s).

### LNs Positive for Metastases by H&E All Express High Levels of CEACAM5 mRNA

**FIGURE 2.**
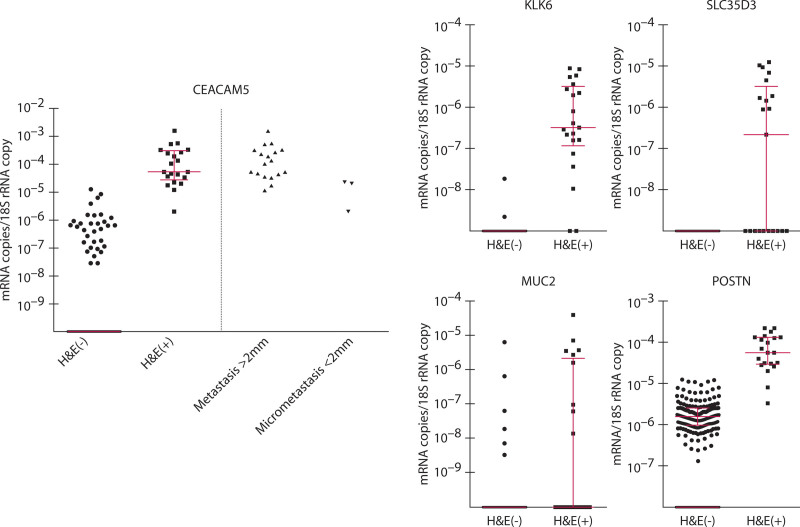
All lymph nodes positive for disseminated tumor cells by microscopic examination of H&E-stained sections had high CEACAM5 mRNA levels. The first of 9 consecutive sections was stained by H&E and examined microscopically for the presence (H&E(+); n = 21) or absence (H&E(–); n = 164) of tumor cells. RNA extracted from the next 8 sections (80-µm section) were analyzed for amounts of CEACAM5, KLK6, SLC35D3, POSTN, and MUC2 mRNAs and 18S rRNA by multiplex qRT-PCR. Expression levels are given as biomarker mRNA copies per 18S rRNA copy. Long, red horizontal bar represents median and the short red horizontal bars represent the 25th and 75th percentile of levels of the indicated biomarker mRNA in H&E(+) and H&E(–) LNs. Each dot represents 1 LN. H&E = hematoxylin-eosin; LN = lymph node; qRT-PCR = quantitative reverse transcription polymerase chain reaction.

Detection of tumor cells in LNs by histopathological examination was compared to estimation of the number of tumor cells by CEACAM5 mRNA levels in 185 LNs from 56 patients with CC. To examine the same region of the LN, 9 consecutive sections were cut. The first was stained by H&E, and RNA was extracted from the remaining 8 sections (80-µm section). Twenty-one of the LNs had detectable metastases (H&E(+)). Eighteen of these had metastases >2 mm and 3 had micrometastases (<2 mm). All H&E(+) LNs had high CEACAM5 mRNA levels (Fig. 2). Lymph nodes with metastases >2 mm had on average higher CEACAM5 levels than those with micrometastases (Fig. 2). However, there was an overlap in that 3 of the LNs with metastases >2 mm had CEACAM5 levels in the range of those of LNs with micrometastases (Fig. 2). Most interestingly, there were 7 H&E(–) LNs that had CEACAM5 levels within the range of H&E(+) LNs (Fig. 2), suggesting that these LNs also contain metastases/micrometastases and should have been classified as H&E(+). An additional 26 LNs had significant CEACAM5 levels, although lower than the H&E(+) LNs. CEACAM5 mRNA was not detected in 131 LNs. The same picture was seen when each patient was represented only by the LN with the highest CEACAM5 level. Thus, the highest LN was H&E(+) in 13 patients, and all had high CEACAM5 levels (Fig. S4 http://links.lww.com/DCR/B649). Among the patients with only H&E(–) LNs, there were 2 patients in whom the LNs had CEACAM5 levels within the range of the H&E(+) LNs, 14 patients with significant but lower CEACAM5 levels, and 27 patients with no detectable CEACAM5 mRNA in their LNs (Fig. S4 http://links.lww.com/DCR/B649).

The other 4 biomarker mRNAs in the combination, ie, KLK6, SLC35D3, POSTN, and MUC2, were all detected, although with different expression patterns (Fig. 2). KLK6 mRNA was detected in the majority of H&E(+) and in 2 H&E(–) LNs. The latter 2 had detectable CEACAM5 mRNA at levels lower than H&E(+) LNs and were the highest LN of 2 patients (Fig. S4 http://links.lww.com/DCR/B649). SLC35D3 mRNA was detected in 11 of the H&E(+) LNs and not in any of the H&E(–) LNs. POSTN mRNA was detected in all but 2 LNs and with significantly higher levels in H&E(+) LNs (median 48.0 × 10^-6^ and 1.5 × 10^–6^ for H&E(+) and H&E(–) LNs; *p* < 0.0001). However, the H&E(–) LNs that had POSTN levels above the 75th quartile all overlapped with the H&E(+) LN POSTN levels, suggesting that as many as 25% of the LNs have signs of influence from disseminated tumor cells. MUC2 mRNA, a marker for good prognosis, was detected in 9 of the H&E(+) and 6 of the H&E(–) LNs.

### Probability of Detecting Aggressive Tumor Cells Increases With the Volume of LN Tissue Analyzed

We investigated the impact of LN tissue volume examined for detection of tumor cells using CEACAM5 mRNA levels as a proxy for tumor cell amounts, and KLK6 and SLC35D3 mRNAs as proxies for tumor cell aggressiveness. Results from 107 half-LN RNA extracts from 30 patients with CC were compared to those from the 80-µm section RNA extracts of the other half of the LN.

**FIGURE 3.**
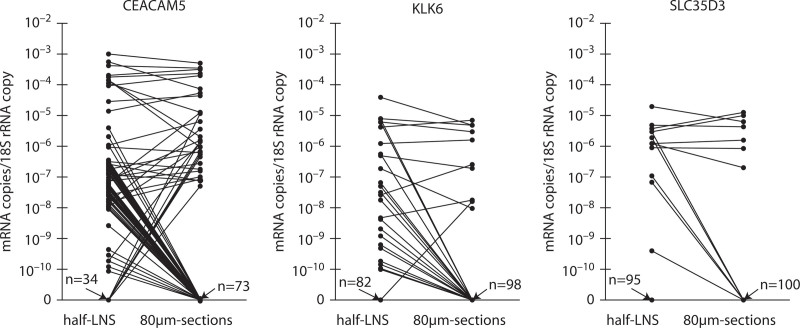
Many CEACAM5-, KLK6-, and SLC35D3-positive lymph nodes are missed in RNA extracted from the small volume of 8 lymph node tissue sections (80-µm sections) compared with RNA extracted from half the lymph node. RNA was extracted from one-half (half-LNs) of 107 LNs and from 8 consecutive, 10-µm-thick sections from the other half of the LN (80-µm sections). Levels, expressed as biomarker mRNA copies per 18S rRNA copy, of CEACAM5, KLK6, and SLC35D3 mRNAs were determined by multiplex qRT-PCR. Each dot represents 1 LN. Lines connect the mRNA levels in half-LN RNA extracts and the corresponding 80-µm section RNA extract of the other half of the LN. n values with arrows pointing to the *x* axis give the number of LNs with no detectable mRNA for the indicated biomarker in half-LN and 80-µm section RNA extracts. LN = lymph node; qRT-PCR = quantitative reverse transcription polymerase chain reaction.

The number of LNs identified as tumor cell positive by detection of CEACAM5 mRNA was significantly increased when the extract was from half-LNs compared with from 80-µm sections (*p* < 0.0001; Fig. 3). CEACAM5 mRNA was detected in 73 half-LN extracts, whereas only 27 of these had detectable CEACAM5 mRNA in the corresponding 80-µm section extracts (Table 2). As many as 46 LNs had detectable CEACAM5 mRNA in half-LNs extracts with no detectable CEACAM5 in the corresponding 80-µm section extract, whereas only 7 LNs had detectable CEACAM5 in the 80-µm sections with no detectable CEACAM5 in the corresponding half-LN extract (Table 2). LNs with high CEACAM5 levels in half-LN extracts had the same or lower levels in the corresponding 80-µm section extracts (Fig. 3). The same results were obtained when patients were represented by the highest LN only. Thus, significantly more patients had CEACAM5 levels in their highest LN that suggested disseminated tumor cells when analysis was done on half-LN extracts compared with 80-µm section extracts (*p* = 0.010; Fig. S5 http://links.lww.com/DCR/B649). The total mRNA copy yield in the half-LN extracts was on average 100-fold higher than the mRNA copy yield in the corresponding 80-µm section extract with a median half-LN extract/80-µm section extract ratio of 96.3 (IQR: 18.9–586).

**TABLE 2.** Importance of LN volume for detection rate of biomarker mRNAs, comparing half-LNs with 80-µm sections

RNA source	CEACAM5(+)^a^		CEACAM5(+)KLK6(+)^a^			CEACAM5(+)SLC35D3(+)^a^		
	n	%^b^	n	CEACAM5(+) LNs,^c^ %	KLK6(+) LNs,^d^ %	n	CEACAM5(+) LNs,^c^ %	SLC35D3(+) LNs,^d^ %
Biomarker profile detected in half-LNs *and* 80-µm sections	27	25.2	8	10.0	33.3	7	8.8	58.3
Biomarker profile detected only in half-LNs	46	43.0	15	18.8	62.5	5	6.3	41.7
Biomarker profile detected only in 80-µm sections	7	6.5	1	1.3	4.2	0	0	0

Half-LNs = half lymph nodes; LN = lymph node; qRT-PCR, quantitative reverse transcription polymerase chain reaction.

a (+), RNA extract was positive for the indicated biomarker mRNA in multiplex qRT-PCR.

b Calculated as % CEACAM5(+) LNs per total number of LNs analyzed, ie, 107 LNs.

c Calculated as % of the 80 LNs that had detectable CEACAM5 mRNA in RNA extracts of fresh-frozen half-LNs and/or formalin-fixed 80-µm sections of the other half of the LN.

d Calculated as % biomarker-positive LNs of total number of biomarker-positive LNs in RNA extracts of half-LNs and/or 80-µm sections of the other half of the LN.

**FIGURE 4.**
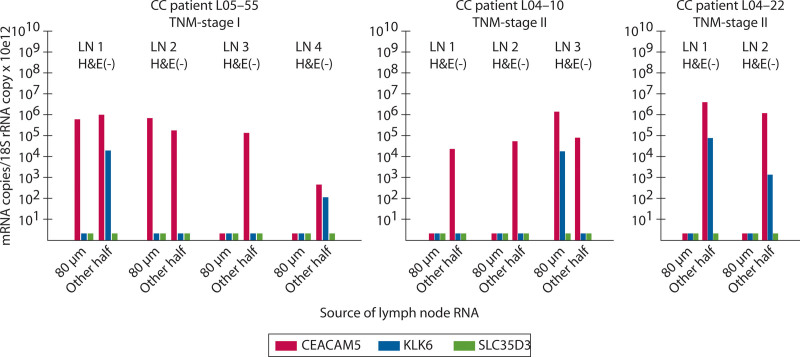
Detection of lymph nodes of patients with stage I and II CC harboring aggressive tumor cells, as indicated by KLK6 mRNA, is increased when RNA is extracted from half a lymph node compared with a few tissue sections. Expression levels of CEACAM5 (red bars), KLK6 (blue bars), and SLC35D3 (green bars) mRNA in 2 to 4 LNs (LN1–LN4) of 1 patient with TNM stage I CC and 2 patients with stage II. Each LN was evaluated for the presence (H&E(+)) or absence (H&E(–)) of disseminated tumor cells by microscopic examination of H&E-stained tissue sections and analyzed for mRNA expression levels in RNA extracted from 80-µm sections adjacent to the section for H&E examination (80 µm) and in RNA extracted from the entire other half of the LN (Other half). Amounts of mRNA and 18S rRNA were determined by multiplex qRT-PCR and results are given as (mRNA copies/18S rRNA copy) × 10^12^. Short bars, below the 10 tick indicate no detection of the mRNA. CC = colon cancer; H&E = hematoxylin-eosin; LN = lymph node; qRT-PCR = quantitative reverse transcription polymerase chain reaction.

**FIGURE 5.**
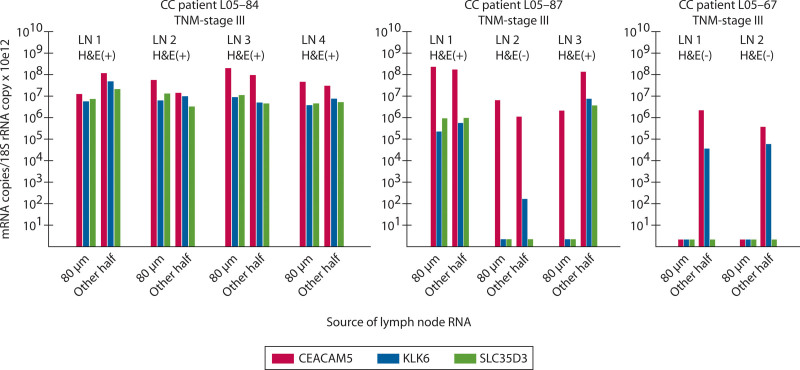
Detection of H&E(–) lymph nodes of patients with stage III CC harboring aggressive tumor cells, as indicated by KLK6 mRNA, is increased when RNA is extracted from half a lymph node compared with a few tissue sections. Expression levels of CEACAM5 (red bars), KLK6 (blue bars), and SLC35D3 (green bars) mRNA in 2 to 4 LNs (LN1–LN4) of 3 patients with stage III disease. Each LN was evaluated for the presence (H&E(+)) or absence (H&E(–)) of disseminated tumor cells by microscopic examination of H&E-stained tissue sections and analyzed for mRNA expression levels in RNA extracted from 80-µm sections adjacent to the section for H&E examination (80 µm) and in RNA extracted from the entire other half of the LN (Other half). Amounts of mRNA and 18S rRNA were determined by multiplex qRT-PCR and results are given as (mRNA copies/18S rRNA copy) × 10^12^. Short bars, below the 10 tick indicate no detection of the mRNA. CC = colon cancer; H&E = hematoxylin-eosin; LN = lymph node; qRT-PCR = quantitative reverse transcription polymerase chain reaction.

KLK6 mRNA was detected in 24 of the LNs. Of these, only 8 LNs had detectable KLK6 in both the half-LN and the 80-µm section extract, 15 LNs had detectable KLK6 only in half-LN extracts, and 1 LN had detectable KLK6 mRNA only in the 80-µm section extract (Fig. 3 and Table 2). Thus, the majority of KLK6-positive LNs are missed due to analyzing the small volume of the 80-µm section extracts. SLC35D3 mRNA showed an expression pattern similar to that of KLK6 mRNA but was detected in fewer LNs (Fig. 3 and Table 2). Figures 4 and 5 show examples of KLK6 and SLC35D3 expression patterns in CEACAM5 mRNA-positive LNs of patients with multiple LNs, comparing 80-µm section and half-LN extracts of the individual LN.

## DISCUSSION

Here we show that: 1) 18S rRNA, measured by qRT-PCR in multiplex and in single-marker format, strongly correlates with the amount of total RNA as determined by OD260 in extracts from both formalin-fixed and fresh-frozen LN tissue, and 18S rRNA is a good “house-keeping gene” for normalization of mRNA expression levels in LNs; 2) determination of CEACAM5 mRNA levels in LN RNA extracts provides an excellent, sensitive assessment of disseminated tumor cells and is more sensitive than histopathological examination of H&E-stained LN tissue for the detection of disseminated tumor cells; 3) increasing the volume of LN tissue from which RNA is extracted strongly increases the sensitivity for detection of tumor cells, measured as CEACAM5 mRNA level, and for the detection of “aggressive” tumor cells expressing KLK6 and/or SLC35D3 mRNAs; and 4) micrometastases are commonly unevenly distributed in LNs.

The utility of determining CEACAM5 mRNA levels to detect disseminated tumor cells is underscored by the fact that all H&E(+) LNs (21/185) in the side-by-side analysis of LN sections by histopathology and qRT-PCR had high CEACAM5 mRNA levels, and several H&E(–) LNs (7/164) had CEACAM5 levels within the same range. Additional H&E(–) LNs (26/164) had lower, but still readily detected CEACAM5 levels. The clinical significance of LNs with CEACAM5 levels below those of H&E(+) LNs is yet to be determined. Approximately one-fourth of the H&E(–) LNs had POSTN mRNA levels in the range of those in H&E(+) LNs, suggesting that these LNs were affected by tumor cells,^14^ which in turn suggests that the LNs with low CEACAM5 levels indeed harbor tumor cells. It is possible that patients with CEACAM5 levels in this lower range, without expressing aggressiveness markers, will, if left untreated, develop distant metastases after a longer time span.

The detection limit for CEACAM5 in the multiplex assay was ≈100 CEACAM5 mRNA copies/µL, which is approximately the signal of 1 tumor cell (range: 30–400 CEACAM5 mRNA copies per cell).^10,11,16^ This, together with the previous finding that very low levels of CEACAM5 mRNA are detected in LNs of control patients,^10,11^ suggests that CEACAM5 mRNA concentrations <100 copies/µL are not relevant for LN classification.

Increasing the tissue volume from which RNA is extracted significantly increased the number of LNs with detectable CEACAM5 mRNA. Thus, more than 40% of the LNs had detectable CEACAM5 mRNA only in the larger volume of a half-LN extract. As expected for unevenly distributed micrometastases, there were occasional LNs (6.5%) in which CEACAM5 mRNA was only detected in the 80-µm section extract. H&E(+) LNs with metastases >2 mm all had significant CEACAM5 levels in extracts from both 80-µm sections and half-LNs. This suggests that metastases >2 mm occupy a large proportion of the LN, thereby making it less important where the sample for analysis is taken from, either for histopathology or CEACAM5 level determination. In contrast, micrometastases ≤2 mm and clusters of tumor cells are unevenly distributed in the LN and the small chance of detection in one or a few sections by histopathology leads to uncertain pN classification. The aggressiveness markers KLK6 and SLC35D3 mRNAs were only detected in CEACAM5-positive LNs. As for CEACAM5, the chance of detecting KLK6 and SLC35D3 increased with increasing LN volume from which RNA was extracted. Over 60% of the KLK6-positive LNs had signals only in the half-LN extract. The corresponding figure for SLC35D3 was over 40%. Because both mRNAs have high prognostic value,^14^ it is important to analyze RNA extracts from as large a volume as possible to identify patients at high risk of recurrence.

**FIGURE 6.**
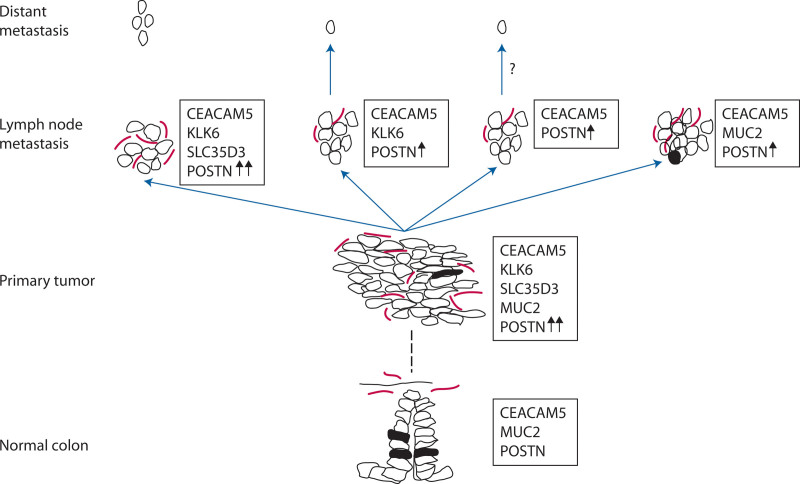
Expression of CEACAM5, KLK6, SLC35D3, POSTN, and MUC2 mRNAs at different stages of tumor development and metastasis in colon cancer: a hypothetical scenario. *Normal colon*: The epithelial cells all express CEACAM5 at the plasma membrane and several of them have differentiated to goblet cells producing mucin-2 (MUC2), the major mucin of colon. Only a few fibroblasts express periostin (POSTN), a ligand for αVβ3 and αVβ5 integrins, to support adhesion and migration of epithelial cells. *Primary tumor*: Epithelial cells that have transformed to cancer cells retain their expression of CEACAM5 and, in varying numbers, also produce MUC2. Fibroblasts in the microenvironment of the tumor cells strongly increase their expression of POSTN, thereby supporting tumor growth. In the growing tumor there is occasional induction of kallikrein-related peptidase 6 (KLK6) expression, that by its proteolytic activity facilitates escape of tumor cells to the draining lymphatics. Expression of solute carrier family 35 member D3 (SLC35D3), an orphan nucleotide sugar transporter with unknown function in the tumor cells is sporadically induced in KLK6-expressing cells. *Lymph node metastasis*: Tumor cells arriving to the lymph node induce elevated POSTN expression in resident fibroblasts, which in turn supports the establishment of micrometastases/metastases, and POSTN expression is increased further with the growing metastasis in a vicious circle. Tumor cells expressing KLK6 are prone to escape also from the metastasis spreading through lymphatics and blood to distant sites where metastases develop. It is possible that tumor cells not expressing KLK6 also leave the lymph node metastasis although likely at a lower frequency. Tumor cells expressing MUC2 are likely to be stationary, with a low degree of proliferation and a high degree of differentiation devoted to mucin production. Open and filled circles = tumor cells and normal epithelial cells; filled circles = MUC2-expressing cells; red elongated structures = POSTN expressing fibroblasts; fat black arrows = increased POSTN expression level; blue arrows = dissemination of tumor cells by lymphatics; hatched line = transformation of colonic epithelial cells to primary colon cancer tumor cells through several steps.

This study shows that the multiplex assay provides accurate determinations of CEACAM5, KLK6, SLC35D3, POSTN, and MUC2 mRNA levels in RNA extracts from tissue volumes as small as a few sections of formalin-fixed tissue, and that analysis of CEACAM5 mRNA in such extracts is a more sensitive technique than histopathology for the detection of disseminated tumor cells. In the present clinical material, the number of tumor cell-positive LNs was 1.33-fold higher based on CEACAM5 mRNA levels than on histopathological examination. Furthermore, we showed that accuracy in detection of tumor cells is strongly improved when the analyzed tissue volume is increased, which is easily achieved with biomarker mRNA analysis but not feasible with histopathology. A possible explanation for why the CEACAM5, KLK6, SLC35D3, POSTN, and MUC2 combination is useful for discriminating between tumors with different propensity to form distant metastasis is shown in Figure 6. To investigate the utility of determining this biomarker combination for predicting the outcome of patients with CRC who had undergone surgical resection, we are currently performing a prospective national multicenter study involving 8 hospitals. In this study *each* LN examined by routine histopathology is also analyzed for biomarker mRNA expression levels. Because the LN volume analyzed is found to increase the detection rate, RNA is extracted from half-LNs in all LNs larger than 2 mm.

## ACKNOWLEDGMENTS

The skillful technical assistance of technologist Assar Bäckman is gratefully acknowledged.

## Supplementary Material


