# Supplementary material for: CEACAM5, KLK6, SLC35D3, POSTN, and MUC2 mRNA Analysis Improves Detection and Allows Characterization of Tumor Cells in Lymph Nodes of Patients Who Have Colon Cancer
Source: Dis Colon Rectum. 2021 Jun 23;64(11):1354–63. doi: 10.1097/DCR.0000000000002151 (PMC8492186; doi:10.1097/DCR.0000000000002151)
Supplement: Supplementary file 1 [file dcr-64-1354-s001.pdf]

Olsson et al. CEACAM5, KLK6, SLC35D3, POSTN and MUC2 mRNA analysis improves detection and allows characterization of tumor cells in lymph nodes of colon cancer patients

APPENDIX

Supplementary Materials and Methods

RNA preparation from formalin-fixed paraffin-embedded lymph node sections

Total RNA was extracted from eight consecutive 10-µm formalin-fixed paraffin-embedded lymph node sections by using the RNeasy FFPE Kit (Cat No: 73504; Qiagen, Sollentuna, Sweden) with the following minor modifications of the manufacturer's protocol: Step 6, PKD-buffer volume increased to 300 µl; Step 15, RBC-buffer volume added increased to 640 µl and sample divided into two tubes; Step 16, 720 µl ethanol added/tube; Step 17, total amount of sample volume from both tubes are transferred in batches of 700 µl to one Rneasy Minelute spin column, centrifuged and flow-through discarded; Step 20, one extra wash with 500 µl RPE.

Supplementary Table S1. Yields of RNA from fresh-frozen half-LNs and formalin-fixed 80µm-sections estimated as total RNA measured by OD260 and 18S rRNA measured by qRT-PCR with an RNA copy standard

RNA source	n[a]	Assay-type	Yield of total RNA and 18S rRNA copies per sample		
			Median	IQR[b]	Range
80µm-sections[c]	200	OD260 measurement by NanoDrop[e] (µg total RNA)	2.9	1.3 - 6.3	0 - 59.4
		Single-marker qRT-PCR assay[f] (18S rRNA copies)	$1.43x10^{10}$	$0.49x10^{10}$-$3.57x10^{10}$	$3.85x10^{7}$ - $111.2x10^{10}$
		Multiplex qRT-PCR assay[g] (18S rRNA copies)	$3.13x10^{10}$	$1.42x10^{10}$ -$6.31x10^{10}$	$2.87x10^{7}$ - $84.25x10^{10}$
Half -LNs[d]	107	OD260 measurement by NanoDrop[h] (µg total RNA)	3,574	1,598 - 17,320	13 - 313,400
		Multiplex qRT-PCR assay[i] (18S rRNA copies)	$1.09x10^{13}$	$0.63x10^{13}$ - $1.84x10^{13}$	$1.07x10^{11}$ - $36.80x10^{13}$

[a] n=number of lymph nodes analyzed.
[b] IQR=interquartile range from the 25th to the 75th percentile.
[c] RNA extracted from eight consecutive sections of formalin-fixed, paraffin-embedded archived lymph nodes.
[d] RNA extracted from fresh-frozen half lymph nodes stored at -70° C until extraction.
[e] Optical density of undiluted RNA extract at 260, 280 and 230 nm determined by NanoDrop and RNA concentration and purity calculated by the built-in formulas.
[f] Concentration of 18S rRNA copies was determined in a 1:100 dilution of the RNA extract by an 18S rRNA-specific qRT-PCR with serial dilutions of an RNA copy standard included in each qRT-PCR run.
[g] Concentration of 18S rRNA copies was determined in undiluted RNA extract using ColoNode® Multiplex qRT-PCR assay designed for determination of concentrations of 18S rRNA, POSTN mRNA and MUC2 mRNA copies in the same reaction mixture.
[h] Optical density of a 1:10 dilution of the RNA extract at 260, 280 and 230 nm determined by NanoDrop and RNA concentration and purity calculated by the built-in formulas.
[i] Concentration of 18S rRNA copies was determined in a 1:10 dilution of the RNA extract using ColoNode® Multiplex qRT-PCR assay.

Supplementary Table S2. Features of the qRT-PCR assays for CEACAM5, KLK6, and MUC2 mRNAs and 18S rRNA

RNA species reverse transcribed and amplified[a]	Forward primer[b]	Reverse primer[b, c]	Probe[d]
CEACAM5 mRNA	5´-CTGATATAGCAGCCCTGGTGTAGT-3´	5´-TGTAGCTGTTGCAAATGCTTTAAG-3´	5´-FAM-AGGAAGACTGACAGTTGT-MGB-3´
KLK6 mRNA	5´-CTTATCCATCCACTGTGGGTC-3´	5´-AAGGTTATGCTTCCCCAGG-3´	5´-FAM-CACTGCAAAAAACCGAATCTTCAGGTC-TAMRA-3´
MUC2 mRNA	5´-AAGAGCGATGCCTACACCAAA-3´	5´-TAGTGTCCAGCTCCAGCATGA-3´	5´-FAM-TCCCGGTTCCACATGA-MGB-3´
18S rRNA	5´-GTAATTCCAGCTCCAATAGCGTA-3´	5´-CGCTCCCAAGATCCAACTAC-3´	5´-FAM-CTGCAGTTAAAAAGC-MGB-3´

[a] The RT-PCR profile was: 50°C for 2 min, 60°C for 30 min, 95°C for 5 min, followed by 45 cycles of 95°C for 20 sec and 60°C for 1 min.
[b] The RNA copy standard covered the amplicon including the primer-sequences. Copy standards were custom synthesized at Dharmacon (Lafayette, CO, USA).
[c] The reverse primer was used as template for reverse transcription in one step qRT-PCR as described (Öberg Å, et al. *Int J Cancer* 2004;111:101–110; Ohlsson L, et al. *Br J Cancer* 2006;95:218-225).
[d] The probe was placed over an exon-boundary in the amplicon.

Supplementary Figure S1

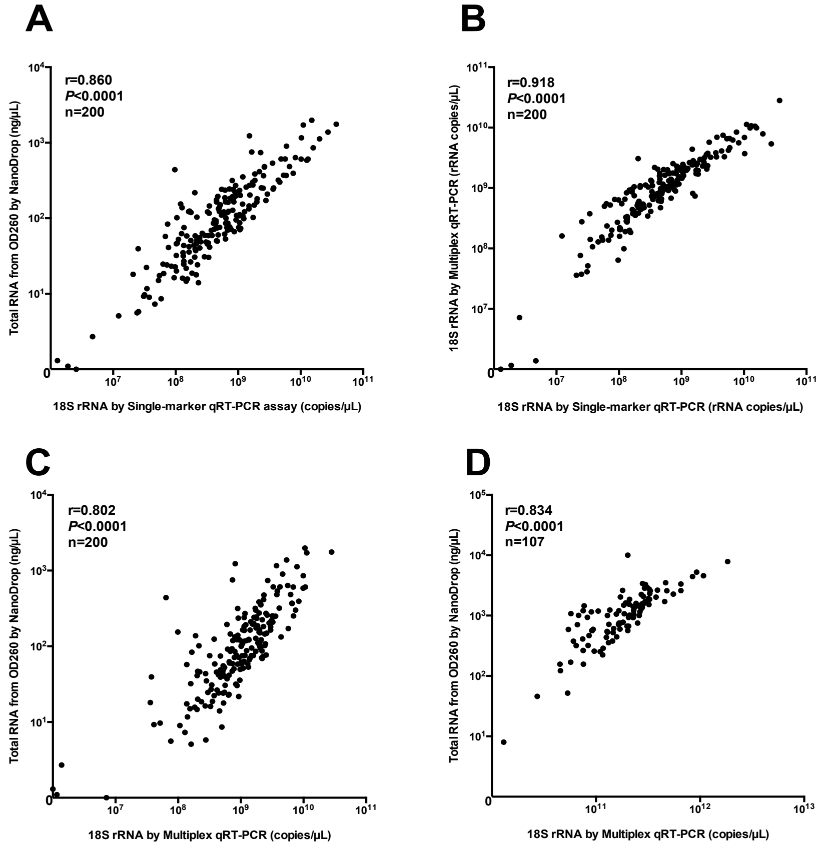

Supplementary Figure S1. ***18S rRNA, determined by both Multiplex- and Single-marker qRT-PCR, gives an excellent estimation of RNA content in lymph node tissue extracts.***
Concentrations of total RNA (ng/µL) measured as OD260 by NanoDrop versus concentration of 18S rRNA (copies/µL) measured by Single-marker qRT-PCR of RNA extracted from 80µm-sections of 200 formalin-fixed, paraffin-embedded LNs. **(B)** Concentrations of 18S rRNA (copies/µL) measured by Multiplex- versus Single-marker qRT-PCR of RNA extracted from 80µm-sections of 200 formalin-fixed, paraffin-embedded LNs. **(C)** Concentrations of total RNA (ng/µL) measured as OD260 by NanoDrop versus concentration of 18S rRNA (copies/µL) measured by Multiplex qRT-PCR of RNA extracted from 80µm-sections of 200 formalin-fixed, paraffin-embedded LNs. **(D)** Concentrations of total RNA (ng/µL) measured as OD260 by NanoDrop versus concentration of 18S rRNA (copies/µL) measured by Multiplex qRT-PCR in RNA extracted from 107 fresh-frozen LN-halves.
r and *P*: correlation coefficient (r) and *p*-value from two-sided Spearman rank correlation analysis. n: number of samples. Each dot represents one LN.

Supplementary Figure S2

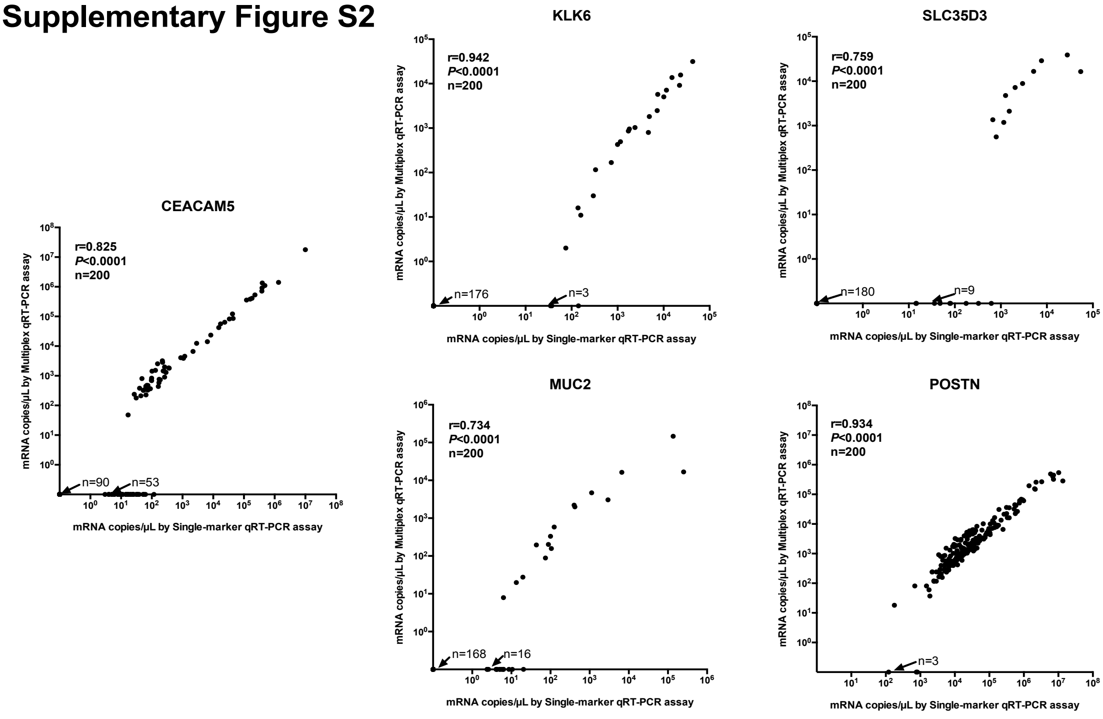

Supplementary Figure S2. ***There is good correlation between concentrations of CEACAM5, KLK6, SLC35D3, POSTN and MUC2 mRNAs determined by Multiplex and Single-marker qRT-PCR analysis*.**
Concentrations of mRNA for CEACAM5, KLK6, SLC35D3, POSTN and MUC2 in RNA extracted from 80µm-sections of 200 formalin-fixed, paraffin-embedded LNs determined by Multiplex qRT-PCR (y-axis) and Single-marker qRT-PCR (x-axis). Results are given as mRNA copies/µL readings from the RNA copy standard run in parallel to the LN samples in the respective assay.
r and *P*: correlation coefficient (r) and *p*-value from two-sided Spearman rank correlation analysis. n: number of samples included in correlation analysis. n with an arrow pointing to origo: number of samples that were negative in both qRT-PCR assays. n with an arrow pointing to the x-axis: number of samples that were positive in Single-marker qRT-PCR assay only. Each dot represents one LN.

Supplementary Figure S3

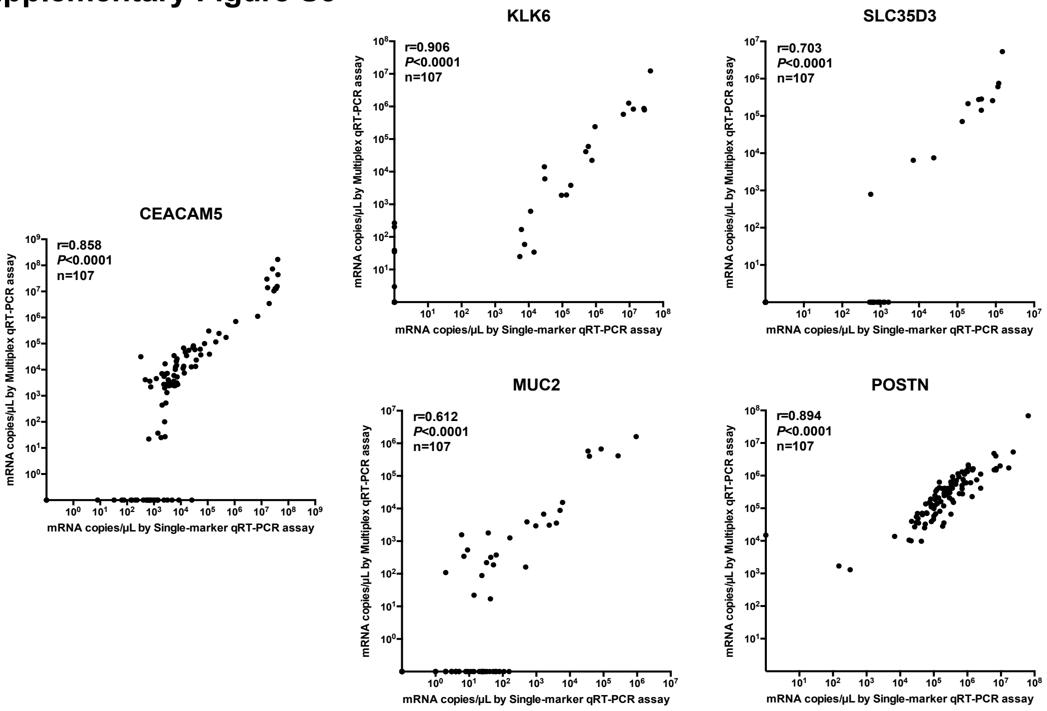

Supplementary Figure S3. ***There is good correlation between concentrations of CEACAM5, KLK6, SLC35D3, POSTN and MUC2 mRNAs determined by Multiplex and Single-marker qRT-PCR analysis***.

Concentrations of mRNA for CEACAM5, KLK6, SLC35D3, POSTN and MUC2 in RNA extracted from half fresh-frozen LNs determined by Multiplex qRT-PCR (y-axis) and Single-marker qRT-PCR (x-axis). Results are given as mRNA copies/µL readings from the RNA copy standard run in parallel to the LN samples in the respective assay.

r and *P*: correlation coefficient (r) and *p*-value from two-sided Spearman rank correlation analysis. n: number of samples included in correlation analysis. Each dot represents one LN.

Supplementary Table S3. Correlation between biomarker mRNA concentrations determined by Multiplex- compared to Single-marker qRT-PCR assay in samples positive in the Multiplex assay

Biomarker mRNA		Correlation between concentration determined by Multiplex- versus Single-marker qRT-PCR[a]	
	n[b]	r-value	*p*-value
CEACAM5	57	0.963	<0.0001
KLK6	21	0.988	<0.0001
SLC35D3	11	0.909	0.0003
POSTN	197	0.931	<0.0001
MUC2	16	0.968	<0.0001

[a] r-values and *p*-values from two-sided Spearman rank correlation test.

[b] Number of LN samples included in the correlation analysis. RNA was extracted from 80μm-sections of 200 formalin-fixed, paraffin-embedded LNs. The table presents results from the LN samples that were positive for the indicated biomarker mRNA in Multiplex qRT-PCR analysis.

Supplementary Figure S4

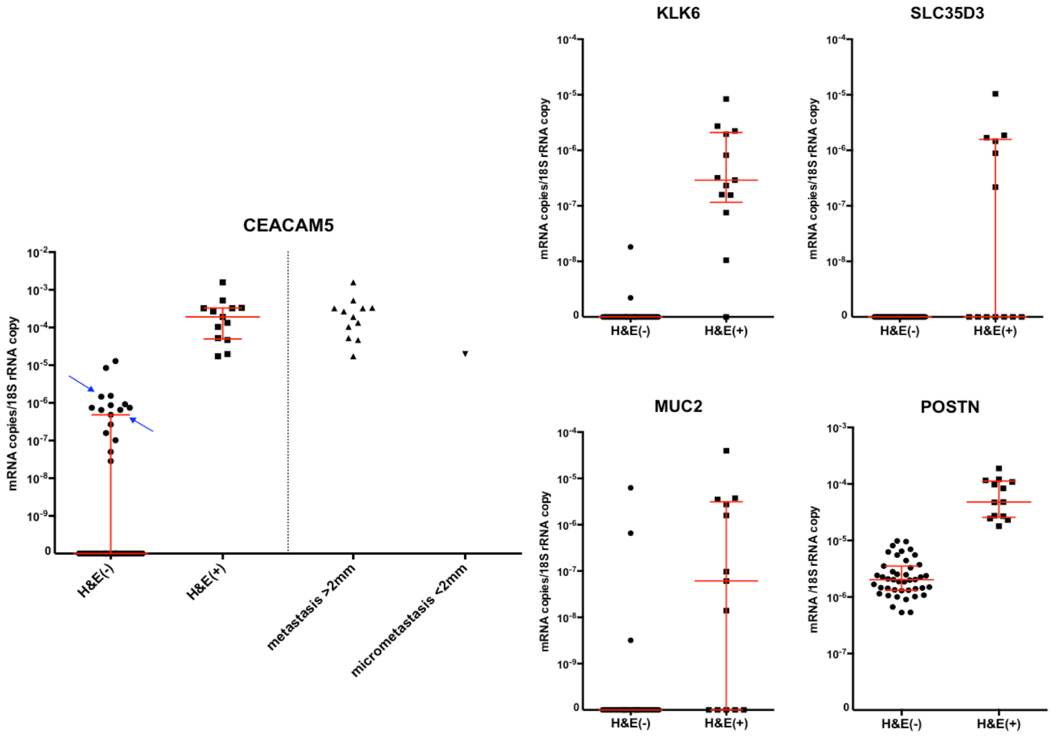

Supplementary Figure S4. ***Comparison between histopathology and biomarker expression based on the lymph node with the highest CEACAM5 level of each patient.***
The first of nine consecutive sections, was stained by H&E and examined microscopically for presence (H&E(+)) or absence (H&E(-)) of tumor cells. RNA extracted from the next 8 sections (80µm-section) were analyzed for amounts of CEACAM5, KLK6, SLC35D3, POSTN and MUC2 mRNAs and 18S rRNA by Multiplex qRT-PCR. For each patient (n=56) the lymph node with the highest CEACAM5 mRNA level was selected and patients were grouped according to the result of histopathology examination, H&E(+) (n=13) or H&E(-) (n=43). Levels of KLK6, SLC35D3, POSTN and MUC2 in the lymph node with the highest CEACAM5 level are also shown. Levels are given as biomarker mRNA copies/18S rRNA copy. Long, red horizontal bar represents median and short, red horizontal bars represent the 25th and 75th percentile of levels of the indicated biomarker mRNA in H&E(+) and H&E(-) LNs, respectively. Blue arrows indicate the two KLK6 mRNA expressing H&E(-) LNs displayed according to their CEACAM5 mRNA level.

Supplementary Figure S5

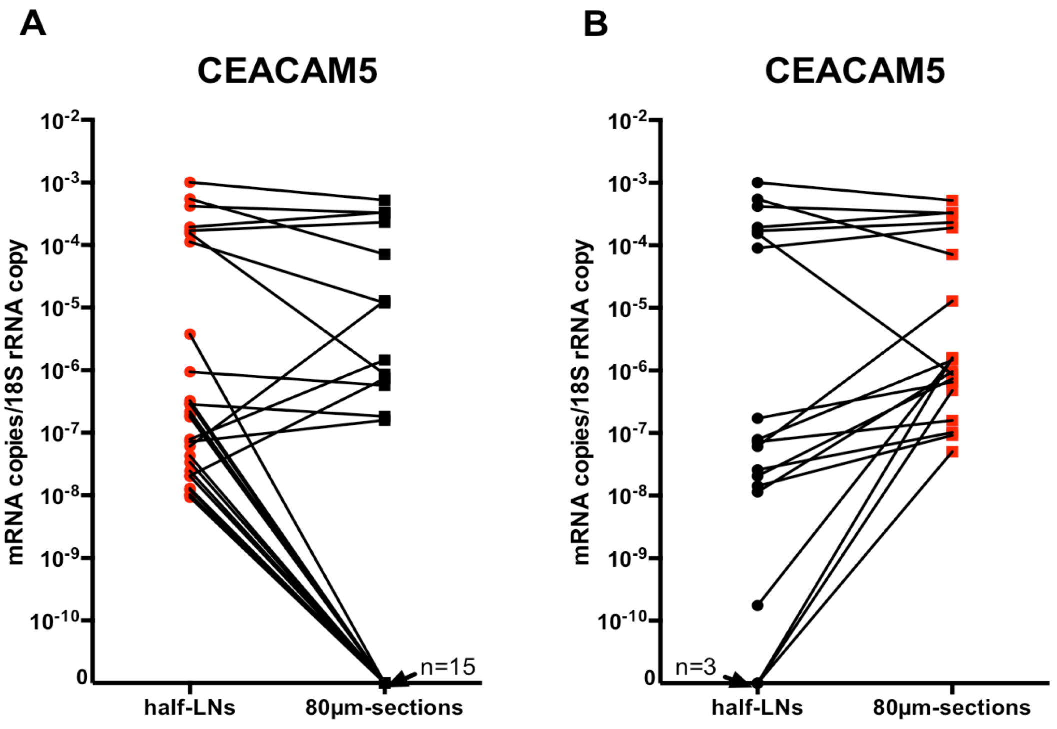

Supplementary Figure S5. ***Significantly more patients show CEACAM5 levels indicating disseminated tumor cells when analysis is done on RNA extracted from half the lymph node compared to the small volume of eight lymph node tissue sections (80µm-sections)***. RNA was extracted from one half (half-LNs) of 107 LNs of 30 CC patients and from eight consecutive, 10 µm thick sections from the other half of the LN (80µm-sections) and analyzed for levels of CEACAM5 mRNA by Multiplex qRT-PCR. CEACAM5-positive LNs were detected in 28 patients when analysis was done on half-LN extracts and in 19 patients when analysis was done on 80µm-section extracts. Each patient with CEACAM5-positive LNs is represented by the LN with the highest CEACAM5 level in half-LN extracts in (**A**) and by the LN with the highest CEACAM5 level in 80µm-section extracts in (**B**). Lines connect the CEACAM5 levels in half-LN RNA extracts and the corresponding 80µm-section RNA extract of the other half of the LN. n-values with arrows pointing to the x-axis give the number of LNs with no detectable CEACAM5 mRNA in 80µm-section extracts (**A**) and half-LN extracts (**B**).
